# Social Interactions in Everyday Life of Socially Anxious Adolescents: Effects on Mental State, Anxiety, and Depression

**DOI:** 10.1007/s10802-023-01121-5

**Published:** 2023-09-28

**Authors:** Julia Ernst, Frank Rückert, Theresa Magdalena Ollmann, Catharina Voss, Hanna Kische, Susanne Knappe, Katja Beesdo-Baum

**Affiliations:** 1 Behavioral Epidemiology, Institute of Clinical Psychology and Psychotherapy, TUD Dresden University of Technology, Chemnitzer Straße 46, 01187 Dresden, Germany; 2 Evangelische Hochschule Dresden (ehs), University of Applied Sciences for Social Work, Education and Nursing, Dresden, Germany

**Keywords:** Social anxiety, Depression, Adolescents, Young adults, Ecological momentary assessment, Everyday life

## Abstract

Unfavorable interpersonal behavior in social anxiety disorder (SAD) contributes to the maintenance of the disorder and may also be related to the development of secondary depression. Since there is limited research on daily life behavior in SAD, this study aimed to describe social interaction behavior and analyze the effect of positive interactions on depression, anxiety, and mental state. Data were obtained from the Behavior and Mind Health study (11/2015–12/2016), an epidemiological cohort study of adolescents and young adults (n = 1,180, aged 14–21 years) from Dresden, Germany. Interpersonal behavior, current mental state, anxiety, and depression were assessed eight times per day over four days using smartphone-based ecological momentary assessments. The analyzed subsample consisted of n = 723 participants, comparing 12-month SAD (n = 60) and healthy controls (HC; n = 663). The interaction behavior of participants with SAD did not differ substantially from that of HC in terms of frequency of social interactions, type of interaction partner, and time spent communicating, although they reported fewer real-life interaction partners (SAD: M = 2.49, SD = 4.78; HC: M = 3.18, SD = 6.43; *F*(17,044) = 23.92, p < 0.001). When comparing mental state, anxiety, and depression after interactions with familiar people to no interaction, no differences were found between SAD and HC. However, interactions with unfamiliar people negatively affected depressive symptoms in individuals with SAD (b = 0.53; *SE* = 0.25; 95%CI: 0.04–1.03;* p* = 0.036). In adolescents with SAD, social situations with unfamiliar people seem to be processed in a dysfunctional way, contributing to increased depressive mood in everyday life. This is particularly interesting given the high rate of secondary depression in SAD.

**Supplementary Information:**

The online version contains supplementary material available at 10.1007/s10802-023-01121-5.

## Introduction

Social anxiety disorder (SAD) is very common in young people (Epkins & Heckler, 2011). The lifetime prevalence rates are estimated to be around 7% in European countries (Fehm et al., 2005), and the highest incidence rates are suggested to be found in childhood and early adolescence (Knappe et al., 2015). Social anxiety is often associated with interpersonal difficulties, including fewer friendships (La Greca & Lopez, 1998; Van Zalk et al., 2011), poorer friendship quality (Biggs et al., 2012; La Greca & Harrison, 2005), poorer social skills (Miers et al., 2010), dysfunctional interpersonal styles (Darcy et al., 2005; Swee et al., 2021), lower levels of assertiveness and greater conflict avoidance (Mufson et al., 2015). Importantly, interpersonal difficulties are associated not only with social anxiety but also with depression (La Greca & Harrison, 2005; Mufson et al., 2015) and have been discussed as an important mediator (Cummings et al., 2014; Erath et al., 2010; Jacobson & Newman, 2016; Mufson et al., 2015; Schleider et al., 2014) in light of the high comorbidity rates (28–50%) of SAD and depression in adolescents (Beesdo et al., 2007; Epkins & Heckler, 2011). Besides bidirectional associations (Belmans et al., 2019), early-onset social anxiety appears to be more predictive of secondary depression than vice versa, making it a relevant risk factor for depression in youth (Beesdo et al., 2007; McLaughlin & King, 2015; Schleider et al., 2014; Van Zalk & Van Zalk, 2019).

Adolescence is a sensitive period in which interpersonal experiences significantly impact affect and behavior. This period is characterized by an increased desire to separate from parents and to gain more autonomy and independence. Peer relationships and close friendships become increasingly important, including social and emotional support (Mufson et al., 2015). However, in socially anxious individuals, safety behaviors and avoidance, as well as anticipatory and post-event processing, not only contribute to the maintenance of anxiety, but also impair effective engagement in social situations and increase the likelihood of negative evaluations by others (Wong & Rapee, 2016). Socially anxious adolescents have been found to have more negative experiences with peers and fewer friends, which can become a vicious cycle that contributes not only to social anxiety but also to depression (Mufson et al., 2015). A cumulative interpersonal risk model suggests that negative peer experiences promote social withdrawal, worry, and rumination, which contribute to loneliness and risk for depression, especially in the absence of close friends (Epkins & Heckler, 2011; Schleider et al., 2014). With this in mind, it is particularly important to examine the interpersonal behavior of people with SAD during the sensitive period of adolescence, when the risk of developing a secondary depression increases (Beesdo et al., 2007).

The everyday interpersonal behaviors of socially anxious adolescents and their impact on mood and depression are best studied using ecologically valid measures of everyday life, such as experience sampling methods (ESM) or ecological momentary assessment (EMA), to minimize retrospective bias and to assess time- and situation-dependent fluctuations (Walz et al., 2014). However, to our knowledge, there has been little research in this area using these methods and, so far depressive mood has not been explicitly considered (Doorley et al., 2020; Goodman et al., 2021; Hur et al., 2019; Morgan et al., 2017). Consistent with retrospective data (Cummings et al., 2014; Mufson et al., 2015), Hur and colleagues (2019) found that socially anxious adolescents had less contact with close companions and exhibited overall higher levels of negative and lower levels of positive affect than non-anxious adolescents. In addition, their EMA results provide information on the variability of affect and show that the social context may have a differential impact on the current affect. Socially anxious adolescents appear to benefit even more from the company of close companions in terms of greater reductions in negative affect, anxiety, and depression than non-socially anxious adolescents (Hur et al., 2019). However, Goodman and colleagues (2021) found contradictory results, with changes in affect across different social situations being quite similar between individuals with SAD and healthy controls. Furthermore, findings from Morgan and colleagues (2017) suggest that emotional closeness with the interaction partner is particularly important for adolescents with SAD. Positive events with less close peers were associated with lower positive affect in youths with SAD compared to healthy youths, whereas positive events with close peers were associated with similar levels of positive affect (Morgan et al., 2017). Besides perceived emotional closeness to interaction partners, the intensity of positive events was found to be an important factor in the emotional benefits of socially anxious people, i.e., the benefits of positive events were greater the more intense these events were rated (Doorley et al., 2020). Taken together, most of these empirical findings suggest that emotional reactivity to everyday events seems to be altered in socially anxious individuals, i.e., they appear to have greater emotional benefits. However, there are also conflicting results and the studies cited used different methods, either testing effects against a continuum of social anxiety or testing people with SAD against healthy controls. Although it is likely that the results would be similar, as people without a diagnosis may also have subthreshold social anxiety, it is important to bear this difference in methodology in mind when interpreting the studies. In addition, most studies used convenience or student samples, which may limit generalizability to other parts of the population, especially since EMA studies are inherently subject to selection bias due to their high demands (Stone et al., 2023), concluding that further research is needed.

In fact, the findings described above partly parallel the mood brightening effect seen in depressive disorders, where people with depression seem to be more responsive to positive events than healthy controls (Khazanov et al., 2019). This means, that higher levels of depression were associated with greater reductions in depressed mood or negative affect after positive events in daily life, especially after positive interpersonal events (Nelson et al., 2020; Panaite et al., 2019; Starr & Hershenberg, 2017). This pattern is explained by the idea that depressed people have fewer positive events in their everyday life, or rate fewer events as positive, resulting in an overall worse mood. A positive event would therefore provide greater contrast and lead to greater reductions in negative affect, also because there is more room for mood improvement (Nelson et al., 2020; Panaite et al., 2018). Comparing these findings to those of social anxiety disorder, it appears that both groups derive greater benefit, i.e. greater mood brightening, from positive (interpersonal) events in daily life than healthy controls, (Hur et al., 2019; Panaite et al., 2018), although this has not been found consistently (Goodman et al., 2021). This potential similarity between SAD and depression may be important given the high comorbidity (Beesdo et al., 2007), as interpersonal difficulties and specific processing of social situations may contribute to secondary depression in SAD.

Against this background, the current study aims to describe the social interaction behavior in the daily life of adolescents and young adults with SAD and to analyze the effect of positive interactions on depression, anxiety, and mental state in terms of a brightening effect. The present study focuses on young people from the general population who meet the criteria for SAD, which seems relevant given the high prevalence of the disorder and its frequent comorbidity, especially with depressive disorders (Beesdo et al., 2007; Epkins & Heckler, 2011; Fehm et al., 2005). The following hypotheses were tested: At first, as socially anxious people tend to avoid social contacts (Mufson et al., 2015) and are characterized by impairments in positive and negative affect (Goodman et al., 2021), it was expected that participants with SAD report fewer interactions, poorer well-being and higher depression and anxiety levels on average in daily life than healthy controls. Second, participants with SAD were expected to derive greater benefits of positive meaningful social interactions concerning mental state and depression than healthy controls (Doorley et al., 2020). Third, these effects might be moderated by the type of interaction partner, with greater benefits following positive interactions with close individuals (Hur et al., 2019; Morgan et al., 2017). That is, it is assumed that a positive effect of social interaction, i.e., the benefit of interaction, should be more pronounced after positive interactions with close people than after interactions with distant/mixed people.

## Methods

### Sample and Procedures

Data stem from the baseline assessment of the Behavior and Mind Health (BeMIND) study, a population-based cohort study of adolescents and young adults from Dresden, Germany. Overall, the study aims to gain a better understanding of trajectories, risk and protective factors for mental disorders in adolescents and young adults. The BeMIND study was conducted in accordance with the ethical standards of the 1964 Declaration of Helsinki and its later amendments, and the study protocol was accepted by the ethics committee of the TUD Dresden University of Technology, Germany (EK381102014). A detailed description of the BeMIND study can be found elsewhere (Beesdo‐Baum et al., 2020).

Briefly, a random sample of 14–21-year-olds, stratified by age and sex, was drawn from the population registry of the city of Dresden (Germany) in 2015. Eligible participants were inhabitants who lived in a household in Dresden during the field period (11/2015–12-2016), were 14–21 years old, had sufficient knowledge of German, and were not institutionalized. Invitation letters were sent by the study team to 6,321 individuals and their families, with a maximum of two reminder letters. 14.1% of these individuals were not eligible, mostly because they did not reside under the provided address, leaving 5,428 individuals. Interested individuals were invited to a personal appointment to provide detailed study information and written informed consent/assent; for minors, written informed consent was also obtained from all legal guardians. The assessments were then conducted. 1,180 individuals completed the BeMIND baseline assessment, resulting in a participation rate of 21.7% (cooperation rate 43.4%) (Beesdo‐Baum et al., 2020). Among active refusers, most common provided reasons for non-participation were lack of time and lack of interest. Overall, participation was higher among females and among those with higher education.

The comprehensive baseline assessments consisted of two in-person assessments approximately one week apart containing a standardized diagnostic interview on day 1, an experimental laboratory and biosampling assessment at day 2, and an online questionnaire and EMA assessment in between.

The EMA assessments were conducted on four consecutive days, including two weekdays and the weekend. The questions were presented via a self-developed smartphone app on eight occasions per day, including one in the morning, six assessments throughout the day, and one assessment in the evening. Each assessment contained 203–248 items and their answer took approximately 3 min each. Branching rules were implemented to minimize the study load, i.e., subquestions were only displayed if the parent question was answered in the affirmative. The smartphone app was programmed for each participant individually regarding their daily life routines. Thus, when distributing reminders for assessments throughout the day, anticipated sleep times and times where they did not want to be disturbed were considered. In addition, the participants could postpone each survey 3 times by 5 min or skip it if was not possible to complete it. The mean time difference between the assessments was M = 134.56 min (SD = 69.54).

For the current analysis, n = 723 (thereof n = 413 female) of the total 1,180 participants were included. Inclusion criteria were, first, the diagnostic status (n = 395 excluded), which was assessed with an updated DSM-5 research version (DIA-X-5; Hoyer et al., 2020) of the Munich Composite International Diagnostic Interview (DIA-X/M-CIDI; Wittchen & Pfister, 1997). Only participants who met the criteria for a 12-month diagnosis of social anxiety disorder (SAD) according to DSM-5 criteria (American Psychiatric Association, 2013) and healthy controls (HC) who did not meet the criteria for any DIA-X-5 diagnosis in the past 12 months (including panic disorder, generalized anxiety disorder, social anxiety disorder, agoraphobia, separations anxiety disorder, specific phobia, obsessive–compulsive disorder, trauma-related disorder, somatic symptom disorder, depressive disorder, bipolar disorder, psychotic disorder, eating disorder, substance use disorder, attention deficit hyperactivity disorder, disruptive, impulse-control or conduct disorder) were included. Second, the availability of EMA data was an inclusion criterion. Participants were only included if at least 50% of the EMA assessments were completed (n = 62 excluded). This results in a sample included in the analyses of n = 60 for SAD (thereof n = 51 female) and n = 663 for HC (thereof n = 362 female). The mean EMA compliance, i.e., the proportion of completed EMA assessments, was 85.7% (SD = 12.2) in SAD and 85.2% (SD = 12.5) in HC.

### Measures

*Self-reported sociodemographic information* containing age, sex, nationality education, and living situations were assessed during the standardized computer-assisted personal interview (DIA-X-5; Hoyer et al., 2020). (Biological) sex was coded as male or female, gender identity was not assessed. Nationality was assessed as German or non-German.

*Diagnostic status* was assessed using the DIA-X-5 (Hoyer et al., 2020) a fully standardized computer-assisted interview administered face-to-face by trained clinical (psychological/medical) interviewers. Supporting lists and dimensional symptom scales were applied via tablet computers. The diagnostic test–retest reliability of the DIA-X-5, tested in a convenience sample of adolescents and adults, yielded a Cohen's kappa between 0.70 and 0.85 for most lifetime diagnoses, the kappa for lifetime SAD was 0.29. The lower kappa in SAD was due to discordance in only one criterion in 9 out of 12 discordant cases. The kappa of test–retest reliability of the core (stem) items for lifetime SAD was 0.83 (Hoyer et al., 2020). Reliability of 12-month diagnoses has not been reported, but intra-class correlations (ICC) for time-related information in the DIA-X-5 were very high (for age of recency > 0.90 for most disorders and 0.98 for SAD) (Hoyer et al., 2020). Validity data for the DIA-X-5 are pending; the prior DIA-X/M-CIDI (Wittchen & Pfister, 1997) revealed in a clinical sample good concordance for most diagnoses with clinician-assigned diagnoses (kappa of 0.63–0.96, except for psychotic disorders, 0.21; dysthymia, 0.54; and somatoform disorders, 0.50). Agreement for SAD was 0.80 (Reed et al., 1998).

Regarding *clinical characteristics*, comorbid diagnoses and current severity of social anxiety (SAD-D) and depression (PHQ-9) were assessed during the diagnostic interview. The DSM-5 Disorder-Specific Severity Measure for Social Anxiety Disorder (SAD-D) (Beesdo-Baum et al., 2012; Knappe et al., 2014; LeBeau et al., 2016) measures the severity of social anxiety and refers to the past four weeks and was completed only if the DIA-X-5 stem question for SAD was endorsed and social anxiety and/or avoidance of social situations was reported to have last occurred within the past 12 months. All items were rated on a 5-point Likert-type scale ranging from 0 (“never”) to 4 (“all of the time”). Both a mean score and a categorical severity classification (0–0.5 none, 0.51–1.5 mild, 1.51–2.5 moderate, 2.51–3.5 severe, 3.51–4 extreme) were formed. The SAD-D scale has been proven to be internally consistent in our sample (Cronbach's alpha = 0.88) and in a sample of undergraduates and treatment seeking adults (Cronbach’s alpha ranging from 0.85 – 0.93) and highly correlated with other validated social anxiety measures (LeBeau et al., 2012, 2016). The Patient Health Questionnaire-9 (PHQ-9) (Kroenke et al., 2001) measures depression severity and refers to the past two weeks and was completed by all participants prior to the DIA-X-5 depression section. The items were rated on a 4-point Likert-type scale ranging from 0 (“not at all”) to 3 (“almost every day”). A sum score and likewise a categorical classification (0–4 minimal, 5–9 mild, 10–14 moderate, 15–19 moderately severe, 20–27 severe) were computed. The PHQ-9 has been shown to be internally consistent in our sample (Cronbach's alpha = 0.77) and in adult samples (Cronbach's alpha = 0.86 and 0.89) and to have good criterion and construct validity (strong association with diagnoses of major depression in independent diagnostic interviews and with measures of functional status) (Kroenke et al., 2001; Reich et al., 2018). Comorbid 12-month diagnoses, assessed with the DIA-X-5, were reported for individuals with SAD. This described whether individuals met criteria for another disorder, specifically an anxiety disorder (including panic disorder, generalized anxiety disorder, agoraphobia, separation anxiety disorder and any specific phobia) or a depressive disorder (including major depressive disorder and persistent depressive disorder (dysthymia)), in the past 12 months.

*EMA-measures* included mental state, depressive and anxious symptomatology, frequency, and characteristics of social interactions, including number of interaction partners and minutes of real-life and online communication, as well as quality and interaction partner of the most meaningful interaction. Except for the categorical choice of the interaction partner and frequency, responses were provided by moving a slider along a bar. Raw scores ranged from 0 to 100 and were scaled to 0 to 10. Specifically, mental state was assessed with the short from of the Multidimensional Mood State Questionnaire (MDMQ, Wilhelm & Schoebi, 2007) and refers to the mood at the moment of assessment (“At the moment I feel …”). At the left and right ends of the slider, the minimum/maximum expressions were labeled respectively (e.g., very tired – very awake). The MDMQ consists of three scales, calmness, energy, and valence, which have been shown to have high internal consistency at the between-person level (0.92—0.90) and at the within-person level (0.70—0.77) in a sample of young adults (Wilhelm & Schoebi, 2007). Due to the high correlation of the scales a mean score was calculated for all six bipolar items, which will be referred to as mental state (Hinz et al., 2012). The internal consistency of mental state was good in our sample (Cronbach's alpha ranged from 0.73 to 0.88 depending on the assessment point; across all assessment points 0.82). Anxiety and depression were assessed with the Patient Reported Outcomes Measurement Information System Version 1.0 Short Forms (PROMIS short form v1.0 anxiety 4a and emotional distress-depression 4a) (Pilkonis et al., 2011) and referred to the time since the last assessment (“Since the last beep…”). The left and right ends of the slider were labeled with "never" and "always", respectively, the middle was labeled “sometimes”, but without specifying exact categories. Higher scores indicate more severe anxiety or depression. The PROMIS scales for anxiety and depression have been shown to have good reliability in a population sample and strong correlations with the long form of the scales, as well as with other validated and accepted measures of anxiety and depression, respectively (Cella et al., 2010). Internal consistency of anxiety and depression was acceptable to good in our sample (Cronbach's alpha ranged from 0.56 to 0.83 for anxiety and 0.82 to 0.94 for depression depending on the assessment point; across all assessment points, alpha was 0.71 for anxiety and 0.90 for depression). Regarding social interactions, if participants reported a social interaction since the last assessment, they were asked to select with whom the most meaningful social interaction took place, multiple answers were possible (partner, family/relative, friend, colleague/ classmate, superior, stranger, other). Referring to the study of Hur and colleagues (2019), the partners were categorized in only close (partner, family/relative, friend), only distant (colleague/ classmate, superior, stranger) and mixed/other (others and close and distant partners at the same time). If an interaction was reported, but the type of interaction partner was missing, it was categorized as mixed/other. Afterwards they could rate on four bipolar items how they felt about the interaction (unpleasant – pleasant, distanced – intimate, conflictual – harmonious, factual – emotional). The quality of interaction (QoI) was the mean score across the dimensions of pleasantness (p), intimacy (i), and harmony (h) (*r*_*p*i_ = 0.69; *r*_*ph*_ = 0.72, *r*_*hi*_ = 0.60; each *p* < = 0.01). In our sample, QoI had high internal consistency (Cronbach's alpha ranged from 0.81 to 0.89 depending on the assessment point; across all assessment points 0.86). Based on the bipolarity of the scales, the interactions were dichotomized into positive (QoI > 5) and negative (QoI < = 5) interactions.

Of the total N = 19,719 observations available, morning assessments (N = 2,571) were excluded since the period queried included the night. Additionally, for the multilevel analyses, we excluded observations where information about the occurrence of an interaction (N = 73) or the quality of the interaction (N = 39) was missing, as well as negative interactions (N = 558), since positive effects on mental state, depression, and anxiety were expected only after positive events (Doorley et al., 2020; Morgan et al., 2017). This results in N = 16,478 observations (SAD: N = 1,324; HC: N = 15,154). Missing values in PROMIS scales reduced available observations for anxiety (SAD: N = 1,316; HC: N = 15,108) and depression (SAD: N = 1,318; HC: N = 15,108) analyses. A detailed flowchart can be found in Online Resource 1.

### Statistical Analyses

Statistical analyses were conducted using STATA 17.0 (StataCorp, 2021). For all analyses, sampling weights were applied to ensure that the distribution of sex and age was representative of the target population of 14–21-year-olds living in Dresden. Details on sampling weights can be found elsewhere (Beesdo‐Baum et al., 2020). Descriptive statistics (weighted percent, %w; mean values, M; standard deviation, SD) were provided regarding sociodemographic and clinical characteristics including age, sex, education, living situation, comorbid diagnosis, and PHQ-9 and SAD-D score and categories, separately for those with 12-months SAD and healthy controls; numbers of participants (n) and observations (N) are reported unweighted. Logistic regressions adjusted for age and sex were calculated for each characteristic to examine associations (odds ratios, OR) with 95%-Confidence Intervals (95%CI) for SAD (vs. HC). The alpha level was set a-priori at α = 0.05. We accept a 5% type 1 error rate for each single test as a feature of our study in exchange for a lower type 2 error rate. This approach without alpha correction favors sensitivity over robustness of findings.

Pearson Chi2 tests, Wald tests, and logistic regressions were used to address the first research question on interaction behavior and mood in everyday life. In detail, mental state, depressive and anxious symptomatology, frequency and characteristics of social interactions, including number of interaction partners and minutes of real and online communication, and quality and interaction partner of the most meaningful interaction were compared between the SAD and HC groups.

To address the second and third research questions, i.e., the effects of positive meaningful social interactions in everyday life, multilevel models (MLMs) were performed, as the data have a hierarchical structure (momentary observations nested within individuals). Only positive interactions were included in these analyses. Separate models were run for mental state, depression, and anxiety. Level 1 predictor was the presence of an interaction (model 1) or the type of interaction partner (model 2; close, distant, mixed/other), no interaction served as dummy-coded reference. Level 2 predictor was the assignment to the groups (SAD, HC). Each model included the main effects as well as the interaction effect of the two predictors. All variables, i.e., the presence/type of interaction and the dependent variable, referred to the same time period. Anxiety and depression scores were highly left-skewed, requiring logarithmization of these scores. This changed the scale from 0 – 10 to -6.9 – 2.3. To improve the prediction, the within-person errors (level 1 residuals) were modeled using autoregressive models of the order 1 based on the assumption that the covariance between two measurements is a decreasing function of the time lag between them. That is, within each individual, the residuals of an observation at time *t* were assumed correlated with the residuals at time *t-1*. The quality criteria AIC and BIC improved when using an autoregressive residual structure compared to an independent residual structure (default in STATA 17.0). In all analyses, sampling weights were added as well as age and sex as covariates.

## Results

### Sample Characteristics

In Table 1, sociodemographic and clinical characteristics are presented separately for both groups, 12-months SAD and HC. Female sex (OR = 4.42; 95%CI: 2.08–9.39; *p* < 0.001), older age (OR = 1.13; 95%CI: 1.02–1.26; *p* = 0.024), non-german nationality (OR = 6.24; 95%CI: 1.34–29.02; *p* = 0.020) as well the severity of depression (PHQ-9: OR = 1.48; 95%CI: 1.34–1.63; *p* < 0.001; PROMIS depression: OR = 1.61; 95%CI: 1.55–1.68; *p* < 0.001), anxiety (SAD-D: OR = 16.37; 95%CI: 7.24–36.98; *p* < 0.001; PROMIS anxiety: OR = 1.72; 95%CI: 1.63–1.81; *p* < 0.001) and mental state (MDMQ: OR = 0.78; 95%CI: 0.75–0.81; *p* < 0.001) were associated with SAD. That is, people with SAD were more likely to be female, older, not of German nationality, and to have higher levels of depression and anxiety and poorer mental state. 76.13% of the SAD-group had at least one comorbid 12-months diagnosis. Looking more closely at comorbid anxiety and depression, 49.14% had another anxiety disorder and 22.35% had a depressive disorder. For those with SAD without any comorbid depressive disorder (SADnoDD; n = 46), the mean depression score (PHQ-9) was M = 7.07 (SD = 4.55) and the mean social anxiety score was (SAD-D) M = 0.95 (SD = 0.59). Among those with any comorbid depressive disorder (SADcomDD; n = 14), mean depression (PHQ-9) was M = 10.55 (SD = 3.50) and mean social anxiety (SAD-D) was M = 1.29 (SD = 0.67). Severity of depression, but not social anxiety, was associated with SADcomDD compared to SADnoDD (PHQ-9: OR = 1.22; 95%CI: 1.00–1.48; *p* = 0.045; SAD-D: OR = 2.29; 95%CI: 0.80–6.61; *p* = 0.122). That is, SADcomDD were more likely to have higher depression scores than SADnoDD. In addition, severity of depression and anxiety were associated with SADnoDD compared to HC (PHQ-9: OR = 1.40; 95%CI: 1.26–1.55; *p* < 0.001; SAD-D: OR = 14.22; 95%CI: 5.92–34.12; *p* < 0.001). That is, SADnoDD were more likely to have higher depression and anxiety scores than HC.

**Table 1:** Sociodemographic and clinical characteristics

	**SAD**		**HC**		**SAD vs. HC**			
	n = 60		n = 663					
	M(SD)		M(SD)		OR	95%CI		*p*
	n	%w	n	%w				
**Age**	18.3 (2.0)		17.6 (2.4)		**1.13**	**1.02**	**1.26**	**0.024**
14–17 years	25	31.29	391	45.88	Ref			
18–21 years	35	68.71	272	54.12	**1.85**	**1.06**	**3.24**	**0.030**
**Sex**								
male	9	21.77	301	55.09	Ref			
female	51	78.23	362	44.91	**4.42**	**2.08**	**9.39**	**< 0.001**
**Nation**								
German	57	93.17	650	97.66	Ref			
non-German	3	6.84	13	2.34	**6.24**	**1.34**	**29.02**	**0.020**
**Education**								
low	0	0.00	9	1.56	ommitted			
middle	11	18.44	118	16.48	1.31	0.60	2.84	0.500
high	47	78.43	517	79.74	Ref			
other	2	3.13	19	2.22	1.78	0.41	7.80	0.445
**Living situation**								
with parents	41	59.33	512	67.89	Ref			
alone	4	8.64	58	11.81	0.47	0.14	1.57	0.223
with partner	5	10.55	24	4.89	1.33	0.43	4.10	0.623
other	10	21.47	69	15.41	1.13	0.45	2.83	0.801
**Comorbid diagnosis (12 months)**								
at least one	46	76.13	–	–	–	–	–	–
no	14	23.87	–	–	–	–	–	–
any other anxiety disorder	30	49.14	–	–	–	–	–	–
any depressive disorder	14	22.35	–	–	–	–	–	–
major depression	9	15.80	–	–	–	–	–	–
dysthymia	9	13.35	–	–	–	–	–	–
**PHQ-9 Depression**	n = 59		n = 658					
mean score	7.86 (4.55)		3.13 (2.44)		**1.48**	**1.34**	**1.63**	**< 0.001**
minimal	11	20.26	504	76.87	Ref			
mild	23	44.06	141	21.18	**7.46**	**3.37**	**16.51**	**< 0.001**
moderate	21	30.43	12	1.87	**52.40**	**18.09**	**151.77**	**< 0.001**
moderately severe	3	3.87	1	0.09	**136.96**	**13.79**	**1,360.14**	**< 0.001**
severe	1	1.38	0	0.00	omitted			
**SAD-D Social Anxiety**	n = 60		n = 217					
mean score	1.03 (0.62)		0.43 (0.35)		**16.37**	**7.24**	**36.98**	**< 0.001**
none	14	24.25	154	68.65	Ref			
mild	34	57.92	62	31.07	**5.89**	**2.70**	**12.83**	**< 0.001**
moderate	9	12.80	1	0.28	**173.84**	**16.00**	**1,889.16**	**< 0.001**
severe	3	5.04	0	0.00	omitted			
extreme	0	0.00	0	0.00	omitted			
	n = 60		n = 663					
**MDMQ mental state (EMA)**	6.24 (2.05)		7.05 (1.61)		**0.78**	**0.75**	**0.81**	**< 0.001**
calmness	6.64 (2.45)		7.51 (1.93)		**0.83**	**0.81**	**0.86**	**< 0.001**
energy	5.36 (2.51)		6.03 (2.31)		**0.91**	**0.88**	**0.93**	**< 0.001**
valence	6.73 (2.42)		7.63 (1.87)		**0.81**	**0.79**	**0.84**	**< 0.001**
**PROMIS anxiety (EMA)**	0.93 (1.39)		0.32 (0.67)		**1.72**	**1.63**	**1.81**	**< 0.001**
**PROMIS depression (EMA)**	1.29 (2.04)		0.35 (0.78)		**1.61**	**1.55**	**1.68**	**< 0.001**

*SAD* 12-months social anxiety disorder, *HC* healthy control, *EMA* ecological momentary assessment, *n* number of participants, *%w* weighted percent, *M* mean, *SD* standard deviation, *OR* odds ratio from logistic regressions, adjusted for sex and age, *Ref.* dummy reference, *CI* confidence interval, bold prints indicate statistical significance, p < 0.05; The SAD-D questionnaire was only filled in when the DIA-X-5 stem question for SAD was endorsed and social anxiety and/or avoidance was reported in the past 12 months, explaining the lower n in the HC group. The EMA statistics reported here are on a 0–10 scale and include all situations (positive, negative, no interaction). After logarithmization, the statistics for PROMIS anxiety were M = -2.86 (SD = 2.39) for HC and M = -1.82 (SD = 2.66) for SAD, and for PROMIS depression were M = -2.82 (SD = 2.35) for HC and M = -1.64 (SD = 2.66) for SAD

### Frequencies and Characteristics of Social Interactions

The frequencies of social interactions as reported in the EMA are shown in Table 2. The frequency of communication with others did not differ significantly between the SAD and HC groups (*F*(17,074) = 0.13, *p* = 0.721), but participants with SAD reported a lower average number of interaction partners in real-life than healthy controls (*F*(17,044) = 23.92, *p* < 0.001). No significant group differences were found for the average minutes spent in real-life and online communication and the number of online interaction partners (all p > 0.05).

**Table 2:** Frequencies of social interactions reported during EMA

	**SAD**		**HC**		**SAD vs. HC**		
	n = 60 N = 1,430		n = 663 N = 15,645				
	N	%w	N	%w	F(df) / OR [95%CI]		*p*
**Since the last beep**							
communication with others (in person, by phone, social media) took place	963	65.91	10,326	65.40	0.13 (17,074)		0.721
**the most meaningful interaction partner was**							
only close	789	80.65	8,187	78.60	1.16 (22,571.46)		0.313
only distant	52	5.77	503	5.81			
mixed/other	122	13.58	1,636	15.59			
**the most meaningful interaction was rated as**							
positive	857	90.21	9835	95.69	Ref		
negative	102	9.79	456	4.31	**2.61 [2.07–3.29]**		**< 0.001**
**meaningful interaction partners by quality of interaction**							
**positive interactions**							
only close	718	82.34	7,905	79.76	1.86 (21,359.69)		0.156
only distant	41	5.41	432	5.29			
mixed/other	98	12.25	1,498	14.95			
**negative interactions**							
only close	71	69.32	277	58.32	2.66 (1,102.09)		0.071
only distant	11	9.33	71	17.82			
mixed/other	20	21.35	108	23.86			
	M	SD	M	SD			
minutes real life communication	35.89	54.87	36.12	48.28	0.02 (17,051)		0.893
minutes online communication	5.96	18.72	5.99	15.85	0.00 (17,051)		0.952
number of interaction partners in real life	2.49	4.78	3.18	6.43	**23.92 (17,044)**		**< 0.001**
number of interaction partners online	0.90	2.50	0.97	2.65	1.23 (17,043)		0.267

All data were weighted. *%w* weighted percent, *SAD* 12-months social anxiety disorder, *HC* healthy control, *n* number of participants, *N* number of observations, *M* mean, *SD* standard deviation, *F* test statistic of Wald-test and chi square test, *df* degree of freedom, *OR* odds ratio from logistic regressions, controlled for sex and age, *Ref.* dummy reference; *CI* confidence interval, bold prints indicate statistical significance, p < 0.05. Categorization of interaction partners: only close (partner, family/relative, friend), only distant (colleague/ classmate, superior, stranger) and mixed/other (others and close and distant partners at the same time). The quality of interaction is the mean of pleasantness, intimacy and harmony and was dichotomized in positive (quality > 5) and negative interactions. The information on the occurrence of an interaction is missing for N = 3 observations in SAD and N = 70 observations in HC. The quality rating is missing for N = 4 observations in SAD and N = 35 observations in HC

For the most meaningful interactions, in the SAD as well the HC group, only close interaction partners (SAD: N = 789, 80.65%; HC: N = 8,187, 78.60%) were most frequently reported, followed by mixed/other (SAD: N = 122, 13.58%; HC: N = 1,636, 15.59%) and only distant (SAD: N = 52, 5.77%; HC: N = 503, 5.81%) interaction partners. No meaningful interaction was reported by N = 467 (34.09%) surveys in individuals with SAD and by N = 5,319 (34.60%) in HC. The distribution of meaningful interactions (no interaction, only close, only distant, mixed/other) was not significantly different between the two groups (*F*(3.00, 51,197.35) = 0.82, *p* = 0.481). A more detailed analysis of the mixed/others category showed that these interactions also predominantly involved a close person (SAD: 93.60%; HC: 89.21%). The most meaningful interactions were predominantly positively rated in both groups, but the odds of a negatively rated interaction were higher in the SAD group (OR = 2.61; 95%CI: 2.07–3.29; *p* < 0.001).

### Effects of Meaningful Positive Social Interactions on Depression, Anxiety, and Mental State

**Fig. 1:**
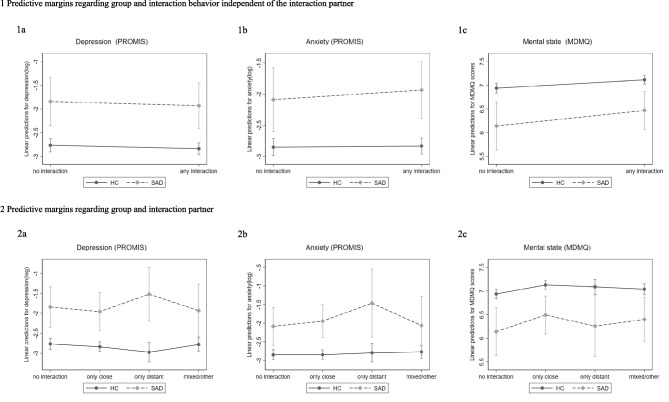
Predictive margins based on multilevel regression models analyzing the effects of positive social interactions on depression, anxiety, and mental state. Parts 1a-1c show predicted depression (1a) anxiety (1b) and mental state (1c) scores related to interaction behaviors for healthy controls (HC) and participants meeting the criteria for 12-months social anxiety disorder (SAD). Parts 2a-2c show predicted depression (2a) anxiety (2b) and mental state (2c) scores for the HC and SAD groups considering the interaction partner. Depression and anxiety scores have been logarithmized. Error bars indicate 95% confidence interval

Table 3 and Fig. 1 parts 1A-1C show the results (coefficients and predictive margins) of the multilevel regression models analyzing the effect of a meaningful positive interaction on depression, anxiety, and mental state, respectively. The interaction effects of group and a social interaction relevant to hypothesis 2 were not significant in any of the models (each p >  = 0.185), indicating that the effect of a positive social interaction did not differ between the SAD and HC groups.

**Table 3:** Multilevel regression models of (1) depression, (2) anxiety, and (3) mental state regarding any positive meaningful interaction

	b	SE	95%CI		*p*
**PROMIS depression**					
sex	**0.53**	**0.12**	**0.30**	**0.77**	**< 0.001**
age	**0.06**	**0.02**	**0.01**	**0.10**	**0.022**
any interaction	-0.07	0.04	-0.16	0.01	0.100
group	**0.92**	**0.27**	**0.40**	**1.44**	**0.001**
any interaction x group	-0.02	0.13	-0.26	0.23	0.897
**PROMIS anxiety**					
sex	**0.46**	**0.12**	**0.22**	**0.70**	**< 0.001**
age	0.03	0.03	-0.02	0.09	0.194
any interaction	0.02	0.04	-0.07	0.10	0.686
group	**0.76**	**0.27**	**0.24**	**1.29**	**0.005**
any interaction x group	0.14	0.16	-0.17	0.45	0.390
**MDMQ mental state**					
sex	**-0.20**	**0.09**	**-0.39**	**-0.02**	**0.034**
age	-0.03	0.02	-0.07	0.01	0.118
any interaction	**0.17**	**0.03**	**0.12**	**0.23**	**< 0.001**
group	**-0.80**	**0.27**	**-1.32**	**-0.28**	**0.003**
any interaction x group	0.15	0.12	-0.07	0.38	0.185

Analyses are adjusted for age and sex (male = 0, female = 1) and considered sample weights and autoregressive models of the order 1. Depression and anxiety scores were logarithmized. Any interaction (no interaction = 0, any interaction = 1), group (healthy control = 0, SAD = 1), *CI* confidence interval, *SE* standard error, bold prints indicate statistical significance, p < 0.05

A significant main effect of group was found in all three models, with individuals with SAD having higher depression scores (b = 0.92; *SE* = 0.27; 95%CI: 0.40–1.44; *p* = 0.001), higher anxiety scores (b = 0.76; *SE* = 0.27; 95%CI: 0.24–1.29; *p* = 0.005) and lower mental state scores (b = -0.80; *SE* = 0.27; 95%CI: -1.32– -0.28; *p* = 0.003) than HC. No main effects of positive social interaction (vs. no social interaction) were found for depression (b = -0.07; *SE* = 0.04; 95%CI: -0.16–0.01; *p* = 0.100) or anxiety (b = 0.02; *SE* = 0.04; 95%CI: -0.07–0.10; *p* = 0.686), but for mental state (b = 0.17; *SE* = 0.03; 95%CI: 0.12–0.23; *p* < 0.001). That is, positive social interactions were associated with more positive mental state scores but not with depression or anxiety.

Table 4 and Fig. 1 parts 2a-2c show the results (coefficients and predictive margins) of the multilevel regression models on meaningful positive interactions, considering the type of interaction partner, on depression, anxiety, and mental state. For depression, the interaction effect relevant to hypothesis 3, close partner by group, was not significant (b = -0.04; *SE* = 0.13; 95%CI: -0.30–0.21; *p* = 0.734), arguing against a brightening effect in individuals with SAD with respect to depressive mood. In contrast, there was a significant interaction effect between only distant interaction partner and group (b = 0.53; *SE* = 0.25; 95%CI: 0.04–1.03; *p* = 0.036). That is, the difference in depression scores between interaction with distant partners and no interaction was higher in individuals with SAD than in HC (see Fig. 1, part 2a). A main effect of group was found, indicating higher scores in individuals with SAD (b = 0.92; *SE* = 0.26; 95%CI: 0.40–1.44; *p* = 0.001) and a tendency towards a main effect of only distant interaction partner was visible, but not significant (b = -0.21; *SE* = 0.11; 95%CI: -0.43–0.01; *p* = 0.065), all other effects were *p* >= 0.105.

**Table 4:** Multilevel regression models of (1) depression, (2) anxiety, and (3) mental state regarding the interaction partner of positive meaningful interactions

	b	SE	95%CI		*p*
**PROMIS depression**					
sex	**0.53**	**0.12**	**0.30**	**0.77**	**< 0.001**
age	**0.06**	**0.02**	**0.01**	**0.10**	**0.019**
only close	-0.07	0.04	-0.16	0.02	0.105
only distant	-0.21	0.11	-0.43	0.01	0.065
mixed/other	-0.02	0.07	-0.15	0.12	0.818
group	**0.92**	**0.26**	**0.40**	**1.44**	**0.001**
only close x group	-0.04	0.13	-0.30	0.21	0.734
only distant x group	**0.53**	**0.25**	**0.04**	**1.03**	**0.036**
mixed/other x group	-0.07	0.27	-0.60	0.45	0.781
**PROMIS anxiety**					
sex	**0.46**	**0.12**	**0.22**	**0.70**	**< 0.001**
age	0.03	0.03	-0.02	0.09	0.194
only close	0.00	0.04	-0.08	0.09	0.941
only distant	0.05	0.11	-0.17	0.27	0.628
mixed/other	0.08	0.07	-0.06	0.22	0.258
group	**0.77**	**0.27**	**0.24**	**1.29**	**0.004**
only close x group	0.14	0.16	-0.17	0.44	0.388
only distant x group	0.57	0.45	-0.31	1.45	0.206
mixed/other x group	-0.06	0.34	-0.72	0.61	0.869
**MDMQ mental state**					
sex	**-0.20**	**0.09**	**-0.39**	**-0.02**	**0.032**
age	-0.03	0.02	-0.07	0.01	0.117
only close	**0.19**	**0.03**	**0.13**	**0.24**	**< 0.001**
only distant	**0.15**	**0.07**	**0.01**	**0.28**	**0.030**
mixed/other	**0.10**	**0.05**	**0.01**	**0.19**	**0.028**
group	**-0.80**	**0.26**	**-1.32**	**-0.28**	**0.003**
only close x group	0.16	0.12	-0.07	0.39	0.163
only distant x group	-0.03	0.26	-0.54	0.48	0.901
mixed/other x group	0.16	0.16	-0.14	0.47	0.302

Analyses are adjusted for age and sex (male = 0, female = 1) and considered sample weights and autoregressive models of the order 1. Depression and anxiety scores were logarithmized. interaction (no interaction = 0, interaction = 1), group (healthy control = 0, SAD = 1), *CI* confidence interval, *SE* standard error, bold prints indicate statistical significance, p < 0.05

Regarding anxiety, a significant group effect was found, indicating higher anxiety scores in individuals with SAD than in HC (b = 0.77; *SE* = 0.27; 95%CI: 0.24–1.29; *p* = 0.004). All other effects, including the interaction close partner by group, were not significant (all p >  = 0.206), indicating that the type of interaction partner did not affect anxiety, and this did not differ between the groups. Although no interaction effects were found at the statistical level, the pattern for anxiety was graphically similar to the pattern for depression (see Fig. 1, part 2b).

For mental state, all interaction effects, including the interaction close partner by group relevant to hypothesis 3, were not significant (all p >= 0.163), indicating no differential effect of the interaction partner type on mental state in individuals with SAD compared to HC (see Fig. 1, part 2c). All main effects were significant, indicating that adolescents with SAD had lower, i.e. worse, mental state scores than HC (b = -0.80; *SE* = 0.26; 95%CI: -1.32– -0.28; *p* = 0.003) and that a positive interaction was associated with higher, i.e. better, mental state scores for each interaction partner (only close: b = 0.19; *SE* = 0.03; 95%CI: 0.13–0.24; *p* < 0.001; only distant: b = 0.15; *SE* = 0.07; 95%CI: 0.01–0.28; *p* = 0.030; mixed/others b = 0.10; *SE* = 0.05; 95%CI: 0.01–0.19; *p* = 0.028). No different patterns of results were observed in sensitivity analyses excluding comorbid depressive disorders in individuals with SAD or considering them as covariates.

As age had a significant effect in some of the analyses and the age range of our sample was relatively wide (14–21 years), we also conducted exploratory analyses with age as a moderator. Age was found to have no moderating effect when the interaction partner type was not included (all p > 0.435). There was also no moderating effect for depression and mental state when interaction partner type was included (all *p* > 0.445). For anxiety, no moderating effects of age were found for distant (*p* = 0.383) or close (*p* = 0.783) interaction partners. There was a three-way interaction effect with mixed/other interaction partners (b = -0.45; *SE* = 0.14; 95%CI: -0.71– -0.18; *p* = 0.001), meaning that interactions with mixed/other individuals were associated with higher anxiety, especially in younger individuals with SAD.

### Explorative Analyses on the Effects of Negative Social Interactions

Because of the counterintuitive results for positive interactions, we decided to re-run the analyses for negative interactions. However, the interpretation of the results must take into account the small number of meaningful negative interactions (see Table 1). Results tables and figures are provided in Online Resources 2–4. In short, negative interactions were shown to have a negative effect on anxiety (b = 0.45; *SE* = 0.12; 95%CI: 0.22–0.69; *p* < 0.001), depression (b = 0.79; *SE* = 0.14; 95%CI: 0.52–1.05; *p* < 0.001), and mental state (b = -0.52; *SE* = 0.09; 95%CI: -0.68– -0.35; *p* < 0.001), but these effects did not differ between HC and individuals with SAD (all interaction effects p >  = 0.535). Regarding the type of interaction partner, all interaction effects did not reach significance (all p >  = 0.052), but from visual inspection, there is a trend that anxiety and depression in individuals with SAD were more affected by negative interactions with distant interaction partners than in HC. This was not the case for mental state.

## Discussion

The aim of the current general population study was, first, to describe social interaction behavior in daily life of adolescents and young adults with a social anxiety disorder, second, to analyze the effect of positive interactions on depression, anxiety, and mental state in terms of a brightening effect (Khazanov et al., 2019), and third, to investigate the impact of the type of interaction partner in this regard. The main findings were that the interactional behavior of young people with SAD was not significantly different from that of healthy controls, and that those with SAD had higher levels of anxiety and depression and poorer mental state in daily life. The assumption of a brightening effect after interactions, especially with close interaction partners, could not be supported. Yet, a negative effect of interactions with distant interaction partners on depressive mood was found in individuals with SAD, even though the interactions themselves were positively rated.

Adolescents and young adults with a 12-month diagnosis of SAD showed overall quite similar communication behavior in daily life as healthy control peers without a 12-months diagnosis of a mental disorder, at least concerning the length of communication and number of interaction partners online. However, they reported a fewer number of interaction partners in real-life. The social network size might be important in this regard. It has been found that socially anxious youths identify fewer people as their friends or confidants (Hur et al., 2019; Van Zalk et al., 2011) and are perceived by others as unattractive interaction partners (Creed & Funder, 1998). Therefore, it might be expected that they have fewer people with whom they could easily interact, albeit this does not affect the number of interactions. This means, they would distribute the same number of interactions among a smaller number of people. However, this assumption needs to be tested elsewhere and, in contrast, social anxiety was also found to be associated with social withdrawal and less time with friends (Biggs et al., 2012; Goodman et al., 2021; Hur et al., 2019).

For the most meaningful interactions, the frequencies of the different interaction partners did not differ between those with SAD and healthy controls, indicating some similarity in interaction patterns. The evaluation of these interactions was mostly positive for all, yet socially anxious people were more likely to rate interactions negatively. This finding is consistent with the theory of post-event processing in social anxiety, according to which socially anxious people are more likely to ruminate negatively after social interactions (Dannahy & Stopa, 2007), which could more often lead to devaluation.

As hypothesized and consistent with the literature (Doorley et al., 2020; Goodman et al., 2021), socially anxious adolescents and young adults reported higher levels of anxiety and depression in daily life and poorer well-being. Yet, an unexpected finding was that the mood brightening effect found in other studies for depression (Bylsma et al., 2011; Nelson et al., 2020; Panaite et al., 2019) and social anxiety (Doorley et al., 2020; Hur et al., 2019; Morgan et al., 2017) was not supported by the current data in the way that socially anxious youths would benefit more from positive interactions. Meanwhile, Goodman and colleagues (2021) have not been able to prove this effect in their study either. Apart from the generally poorer well-being and higher levels of depression, and anxiety found in socially anxious young adolescents, positive interactions in daily life instead appear to have overall effects similar to those in non-anxious healthy individuals. However, when the type of interaction partner was considered, interactions with only unfamiliar, distant individuals were found to be associated with higher levels of depression compared to no interaction in socially anxious individuals. Thus, interactions with distant people seem to have a particularly disadvantageous effect. This was most evident for depressive symptoms. But, graphically, a trend also emerged for anxiety. With respect to mental state, no differential effects on the benefit of positive interactions were found, although a tendency for a brightening effect after close or mixed interactions was observed graphically.

There may be several reasons why our study did not find the mood brightening effect, but rather an association of increased depressiveness with social interactions with distant, unfamiliar people. It is important to look at the methodological differences from the studies that found the brightening effect. These studies asked about current or contextual affect (Doorley et al., 2020; Hur et al., 2019; Morgan et al., 2017). For anxiety and depression symptoms, we referred to the time since the last assessment, which was on average more than two hours ago. Given that people with SAD tend to have negative post-event processing (Dannahy & Stopa, 2007), one might assume that positive experiences would be devaluated over time, so that potential brightening effects would only be found during the event. The longer assessment interval left a little more room for retrospective bias, which, combined with the negative post-processing, may have led people with SAD not to report a possible reduction in depression and anxiety symptoms. However, this is contradicted by the study by Goodman and colleagues (2021), who also did not find the effect, despite examining the current affect and context. The evidence for the mood brightening effect in people with social anxiety is thus very heterogeneous, and further studies are needed to identify possible mediator or moderator variables.

Further, it remains to be discussed why social interactions with unfamiliar, distant people were associated with increased levels of depression in youths with SAD, even though these interactions themselves were not rated as negative. Communicating with unfamiliar people is a very uncomfortable situation for individuals with SAD (Ruscio et al., 2008) and can lead to negative self-evaluations and increased rumination afterwards (Dannahy & Stopa, 2007; Kocovski et al., 2005). Referring to Clark and Wells’ (1995) cognitive model, they focus on the negative feelings and cognitions they experienced in the situation and may infer incompetence, leading to self-devaluation and increased feelings of depression. It is now questionable why the situation was however not rated as negative. As a reminder, in this study an interaction was defined as negative if the mean of the bipolar scales pleasantness, intimacy, and harmony was below the cutoff of 5, which is the midpoint of the response scale. Thus, one might assume that socially anxious people evaluated the interaction in two parallel ways: On the one hand, more objectively, in the sense that there were no overt conflicts, and the interaction was generally friendly. On the other hand, they may strongly devalue their own role in the interaction and attribute negative aspects of the situation to themselves, which increases feelings of incompetence and depressive thoughts. Combined, this could indicate an adverse attributional style, similar to that seen in depression, namely internal, stable, and global attributions for negative events (Fresco et al., 2006; Sweeney et al., 1986). This also parallels to the theory of double standard bias in social anxiety, according to which socially anxious individuals tend to make more stringent predictions about themselves than about others (Voncken et al., 2006). However, the assumption of an attributional style that moderates depressive thoughts following interactions with strangers in socially anxious individuals remains to be tested.

One might further assume that, despite the dichotomization of quality and that in this sense only positive interactions were considered, socially anxious individuals might have rated the quality of distant interactions worse than healthy controls, so that the quality rating might act as a mediator. Although this assumption was not fully tested, a closer look at the descriptive data revealed no remarkable differences in the quality ratings between socially anxious and healthy participants. Thus, it appears that factors other than the perceived quality of the interaction contribute to increased depressive feelings following distant interactions in socially anxious individuals.

The increased depressiveness did not occur when familiar people, like friends or family members, were involved in the interactions. This could be explained by the fact that interaction situations with familiar people may not be anxiety-provoking for socially anxious and, when unfamiliar people are also involved in the situation, familiar people might instead serve as regulatory role (Morgan et al., 2017).

It is important to note, that anxiety ratings showed a similar pattern to depression, although the effect did not reach significance, probably due to large variances. Social interactions with unfamiliar people are among the most feared situations in social anxiety (Beidel et al., 2007; Stein et al., 2010). So, one might have expected that the difference in anxiety between interactions with strangers and no interaction would be more pronounced in people with SAD than in healthy people. This tendency can also be seen visually. A quite similar picture can be found for current mental state, which seems to be higher for both groups after interaction situations and in general lower for the socially anxious. Although the interaction effects were not statistically significant, descriptively, there was a higher dynamic between interaction situations and a tendency toward a brightening effect in socially anxious individuals. Unlike other studies that have examined momentary affect as a function of social interactions (Goodman et al., 2021; Hur et al., 2019), we asked about the most meaningful interaction situation since the last assessment, rather than the current social situation. However, the mental status questions referred to the current mental conditions. This is important in that the dynamics of the current affect and differences between individuals with SAD and healthy individuals may be even more pronounced considering the current situation. Furthermore, the exploratory moderation analyses of age suggest that age did not moderate the effect of social interactions on depression and mental state, but partially on anxiety. The results suggest that younger people with SAD seem to feel more anxious after social interactions with mixed groups/other people, which may be related to new social contexts that become particularly relevant in early adolescence (e.g., starting to go out with friends). In conclusion, there is a need for further research on the emotional dynamics of different social situations in in young people with social anxiety disorder.

The exploratory post hoc estimates of the effects of negative interactions showed that negative interactions in daily life were associated with higher levels of depressive and anxiety symptoms and poorer mental state in healthy individuals and individuals with SAD. Interestingly, there may be as well group differences when the type of interaction partner is taken into account. Although not statistically supported due to lack of power, the data graphically suggested that negative interactions with close people had a negative effect for all, whereas negative interactions with distant people seemed to be particularly distressing for the adolescents with SAD. This suggests that healthy individuals may be better able to distance themselves from negative social experiences with unfamiliar people, whereas this does not seem to be the case in adolescents and young adults with SAD. This would support the specific role that interacting with unfamiliar people plays in the daily lives of socially anxious adolescents. Also, findings may likely be explained by cognitive bias and double standards, similar to positive interactions.

There are some limitations to be mentioned. First, the sample size of the SAD group (n = 60) was quite small, and only adolescents and young adults living in the city of Dresden, Germany, participated, which limits the interpretation and generalization of the results to other regions, cultures, or age groups. The results of the moderation analyses by age must be interpreted with caution due to limited power. Moderation analyses by sex were not conducted due to small subsamples, although this would be interesting to consider for further research, as the adjustment appeared to be relevant in some parts of our analyses. We adjusted for age and sex, which allowed the results to be interpreted independently of the expression of these variables. Since this was not a clinical sample and mainly mild forms of social anxiety were included, fear and avoidance behavior were likely to be less pronounced compared to clinical SAD. In addition, the HC group was defined as a very healthy group with no DSM-5 diagnosis in the past 12 months, which may not be considered a typical population reference. However, as social interactions are impaired in various ways in many mental disorders, it would be very difficult to interpret the effect in individuals with SAD against a mixture of all other disorders and healthy people. Thus, our approach allowed us to test the effects for SAD, although no conclusions can be drawn about its specificity or other psychopathologies. Interpretation of the results of the exploratory analyses on negative social interactions is limited due to the small number of meaningful negative interactions reported. Nevertheless, the results can be interpreted as a tendency and provide starting points for further research.

Beside these limitations, there are also strengths to be highlighted. The study examined daily life data from a general population sample of adolescents and young adults, so it can be assumed to have high ecological validity in the context of the groups studied. The diagnostic status was assessed in each participant by means of a fully standardized computer-assisted personal interview and the diagnoses were based on current DSM-5 diagnostic criteria. In light of this, our study provides important information, complementary to previous EMA studies (e.g. Doorley et al., 2020; Goodman et al., 2021; Hur et al., 2019), on social interaction behavior and its emotional impact in young people with mild forms of SAD.

Our study provides directions for future research. In addition to replicating our results, researchers could examine which factors explain the elevated depression scores following contact with unfamiliar people in socially anxious adolescents. Investigating this pattern and potential moderating factors is particularly interesting given the high rates of secondary depression in SAD (Beesdo et al., 2007). Overall, it seems useful to further investigate emotional variability and stability in everyday life of young people with SAD. As our study included only mild forms of SAD, examination of daily life records of people with clinically relevant SAD with and without comorbid depression could provide further important information.

In summary, adolescents and young adults with mild forms of SAD seem to have a quite similar pattern of interaction in daily life as healthy individuals. However, consistent with other studies (Goodman et al., 2021; Hur et al., 2019), our findings suggest that the emotional effects of social interactions are altered. In particular, the type of interaction partner appears to be of greater importance to emotional state in people with SAD than in healthy individuals. That is, we found meaningful interactions with unfamiliar individuals to be associated with increased depression in young people with SAD. Given the high rate of secondary depression in SAD, this is an important indication of potentially problematic cognitive processing of such situations. Accordingly, our results provide an important starting point for further research on the development of depressive symptoms in social anxiety and the potential progression to depressive disorders.

### Supplementary Information

Below is the link to the electronic supplementary material.

Supplementary file1 (DOCX 289 KB)

## Data Availability

The data that support the findings of this study are available from the senior author upon reasonable request.
