# Supplementary material for: Social Interactions in Everyday Life of Socially Anxious Adolescents: Effects on Mental State, Anxiety, and Depression
Source: Res Child Adolesc Psychopathol. 2023 Sep 28;52(2):207–22. doi: 10.1007/s10802-023-01121-5 (PMC10834615; doi:10.1007/s10802-023-01121-5)
Supplement: Supplementary file 1 — Supplementary file1 (DOCX 289 KB) [file 10802_2023_1121_MOESM1_ESM.docx]

**Supplementary Information**

**Assessed BeMIND baseline participants (n=1,180)**

**Excluded (n=457)**

- Not meeting diagnostic inclusion criterion (neither 12-months SAD nor healthy control; n=395)
- No EMA assessment or not a minimum of 50% EMA compliance (n=62)

**Sample included in the analyses (n=723)**

**Healthy control group (n=663)**

- No 12-months DSM 5 diagnosis

**SAD group (n=60)**

- 12-months social anxiety disorder diagnosis

**Total EMA observations available (N=19,719)**

**Multilevel analyses (N=16,478)**

**Healthy control group (N=15,154)**

EMA observations including positive interactions (N=9,835) and no interactions (N=5,319)

- Mental state (MDMQ): available for analysis N=15,154
- Anxiety: available for analysis N=15,108
- Depression: available for analysis N=15,108

**SAD group (N=1,324)**

EMA observations including positive interactions (N=857) and no interactions (N=467)

- Mental state (MDMQ): available for analysis N=1,324
- Anxiety: available for analysis N=1,316
- Depression: available for analysis N=1,318

**Description of social interactions (N=17,075)**

**Excluded (N=2,644)**

- Morning assessment, as the period queried included the night (N=2,571)
- Assessments where information about the occurrence of an interaction was missing (N=73)

**Excluded (N=597)**

- Interactions where the quality rating was missing (N=39)
- Negative interactions, as effects were only expected after positive events (N=558)

**Missing values**

- Anxiety scale (N=8)
- Depression scale (N=6)

**Missing values**

- Anxiety scale (N=46)
- Depression scale (N=46)

*Online Resource 1.* Flowchart of the analysis sample. n, number of participants; N, number of observations; SAD, 12-months social anxiety disorder

| ***Online Resource 2.*** Multilevel regression models of (1) depression, (2) anxiety, and (3) mental state regarding any negative meaningful interaction | | | | | |  |
| --- | --- | --- | --- | --- | --- | --- |
|  |  |  |  |  |  |  |
|  | b | SE | 95%CI | | *p* |  |
| **PROMIS depression** |  |  |  |  |  |  |
| sex | **0.66** | **0.13** | **0.40** | **0.92** | **<.001** |  |
| age | **0.05** | **0.03** | **0.00** | **0.10** | **.069** |  |
| any interaction | **0.79** | **0.14** | **0.52** | **1.05** | **<.001** |  |
| group | **0.83** | **0.27** | **0.29** | **1.36** | **.003** |  |
| any interaction x group | 0.19 | 0.30 | -0.40 | 0.77 | .535 |  |
|  |  |  |  |  |  |  |
| **PROMIS anxiety** |  |  |  |  |  |  |
| sex | **0.50** | **0.14** | **0.22** | **0.77** | **<.001** |  |
| age | -0.01 | 0.03 | -0.07 | 0.05 | .793 |  |
| any interaction | **0.45** | **0.12** | **0.22** | **0.69** | **<.001** |  |
| group | **0.81** | **0.28** | **0.26** | **1.35** | **.004** |  |
| any interaction x group | 0.13 | 0.29 | -0.43 | 0.69 | .654 |  |
|  |  |  |  |  |  |  |
| **MDMQ mental state** |  |  |  |  |  |  |
| sex | **-0.30** | **0.11** | **-0.51** | **-0.08** | **.007** |  |
| age | **-0.05** | **0.02** | **-0.09** | **0.00** | **.042** |  |
| any interaction | **-0.52** | **0.09** | **-0.68** | **-0.35** | **<.001** |  |
| group | **-0.75** | **0.28** | **-1.30** | **-0.19** | **.008** |  |
| any interaction x group | 0.05 | 0.25 | -0.44 | 0.53 | .850 |  |
| *Note.* Analyses are adjusted for age and sex (male=0, female=1) and considered sample weights and autoregressive models of the order 1. Depression and anxiety scores were logarithmized. Any interaction (no interaction=0, any interaction=1), group (healthy control=0, SAD=1); CI, confidence interval; SE, standard error; bold prints indicate statistical significance, p<.05. | | | | | |  |
|  |  |  |  |  |  |  |
|  |  |  |  |  |  |  |
|  |  |  |  |  |  |  |

| ***Online Resource 3.*** Multilevel regression models of (1) depression, (2) anxiety, and (3) mental state regarding the interaction partner of negative meaningful interactions | | | | | |
| --- | --- | --- | --- | --- | --- |
|  |  |  |  |  |  |
|  | b | SE | 95%CI | | *p* |
| **PROMIS depression** |  |  |  |  |  |
| sex | **0.67** | **0.13** | **0.41** | **0.93** | **<.001** |
| age | 0.05 | 0.03 | 0.00 | 0.11 | .049 |
|  |  |  |  |  |  |
| only close | **0.89** | **0.15** | **0.59** | **1.18** | **<.001** |
| only distant | -0.08 | 0.28 | -0.64 | 0.48 | .780 |
| mixed/other | **1.20** | **0.26** | **0.68** | **1.72** | **<.001** |
| group | **0.82** | **0.27** | **0.28** | **1.36** | **.003** |
|  |  |  |  |  |  |
| only close x group | 0.18 | 0.31 | -0.43 | 0.79 | .560 |
| only distant x group | 1.42 | 0.73 | -0.02 | 2.85 | .052 |
| mixed/other x group | -0.75 | 0.67 | -2.05 | 0.56 | .261 |
|  |  |  |  |  |  |
| **PROMIS anxiety** |  |  |  |  |  |
| sex | **0.50** | **0.14** | **0.22** | **0.78** | **<.001** |
| age | -0.01 | 0.03 | -0.07 | 0.05 | .818 |
|  |  |  |  |  |  |
| only close | **0.43** | **0.17** | **0.11** | **0.76** | **.009** |
| only distant | 0.30 | 0.24 | -0.17 | 0.77 | .213 |
| mixed/other | **0.62** | **0.20** | **0.22** | **1.01** | **.002** |
| group | **0.81** | **0.28** | **0.26** | **1.35** | **.004** |
|  |  |  |  |  |  |
| only close x group | 0.14 | 0.28 | -0.40 | 0.68 | .618 |
| only distant x group | 0.78 | 0.96 | -1.11 | 2.66 | .419 |
| mixed/other x group | -0.23 | 0.69 | -1.59 | 1.12 | .736 |
|  |  |  |  |  |  |
| **MDMQ mental state** |  |  |  |  |  |
| sex | **-0.30** | **0.11** | **-0.52** | **-0.08** | **.007** |
| age | **-0.05** | **0.02** | **-0.09** | **0.00** | **.039** |
|  |  |  |  |  |  |
| only close | **-0.41** | **0.11** | **-0.64** | **-0.19** | **<.001** |
| only distant | **-0.48** | **0.20** | **-0.86** | **-0.09** | **.015** |
| mixed/other | **-0.77** | **0.17** | **-1.11** | **-0.43** | **<.001** |
| group | **-0.75** | **0.28** | **-1.30** | **-0.19** | **.008** |
|  |  |  |  |  |  |
| only close x group | -0.17 | 0.24 | -0.64 | 0.31 | .488 |
| only distant x group | -0.13 | 0.53 | -1.17 | 0.91 | .804 |
| mixed/other x group | 0.76 | 0.48 | -0.18 | 1.70 | .112 |
| *Note.* Analyses are adjusted for age and sex (male=0, female=1) and considered sample weights and autoregressive models of the order 1. Depression and anxiety scores were logarithmized. interaction (no interaction=0, interaction=1), group (healthy control=0, SAD=1); CI, confidence interval; SE, standard error; bold prints indicate statistical significance, p<.05. | | | | | |
|  |  |  |  |  |  |
|  |  |  |  |  |  |
|  |  |  |  |  |  |

1 Predictive margins regarding group and interaction behavior independent of the interaction partner

1C

1B

1A


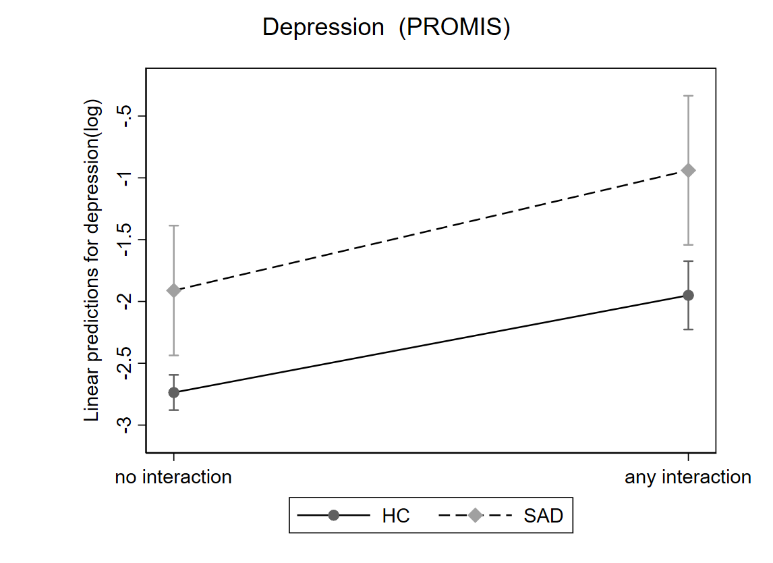

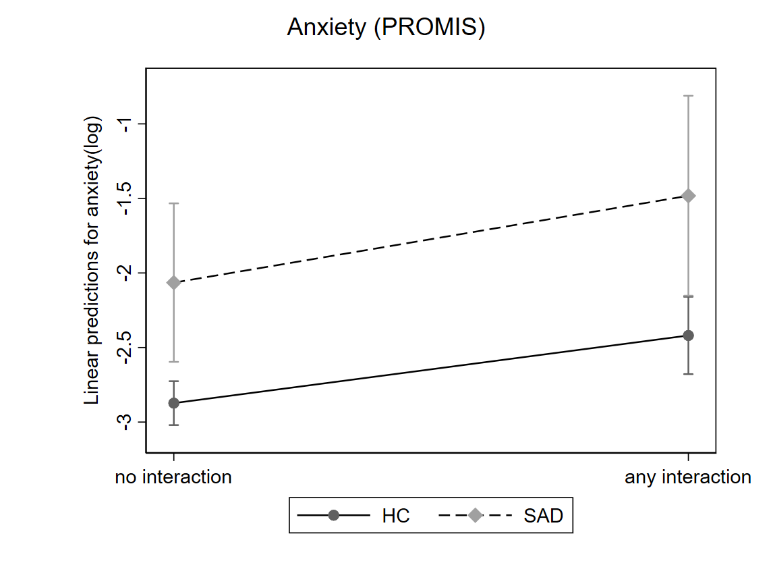

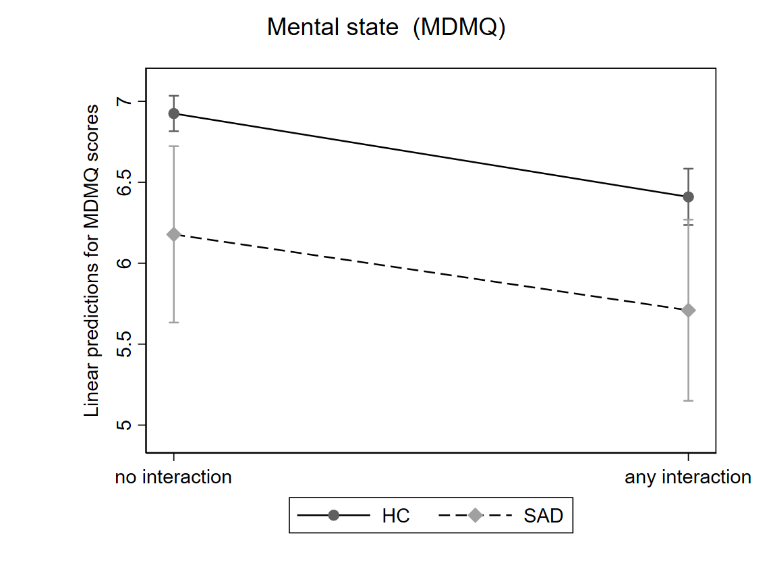


2 Predictive margins regarding group and interaction partner

2C

2B

2A


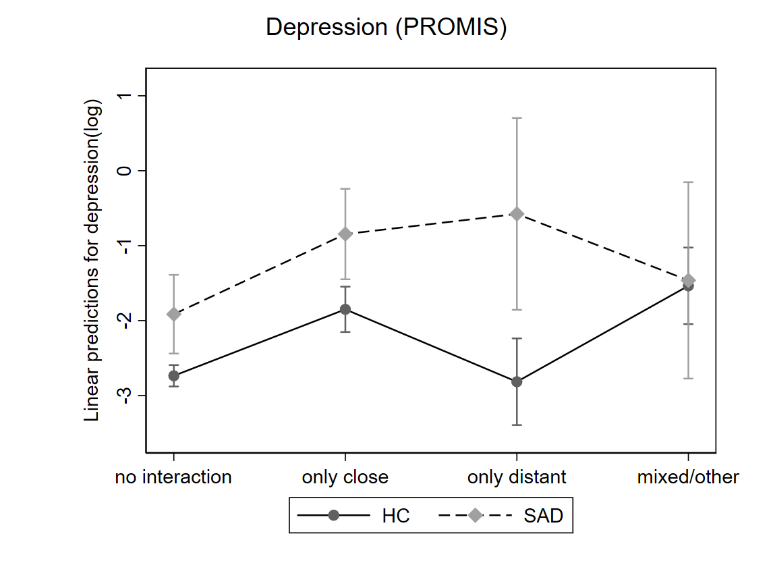

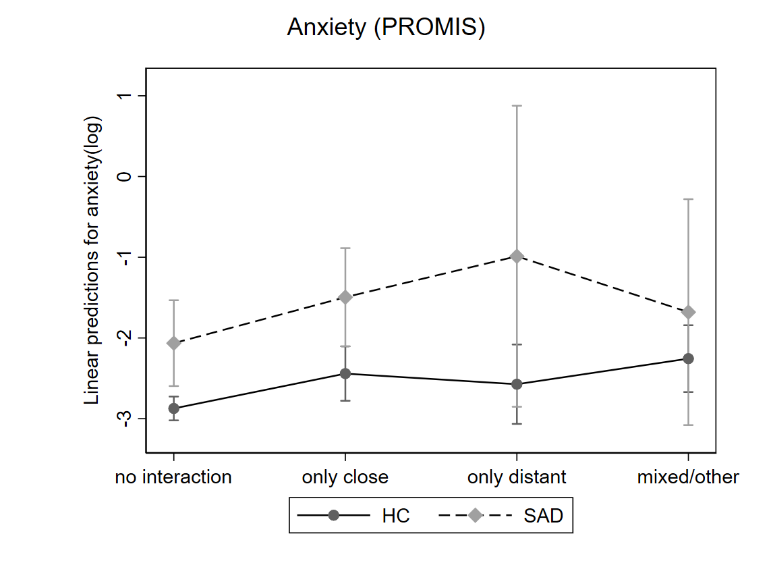

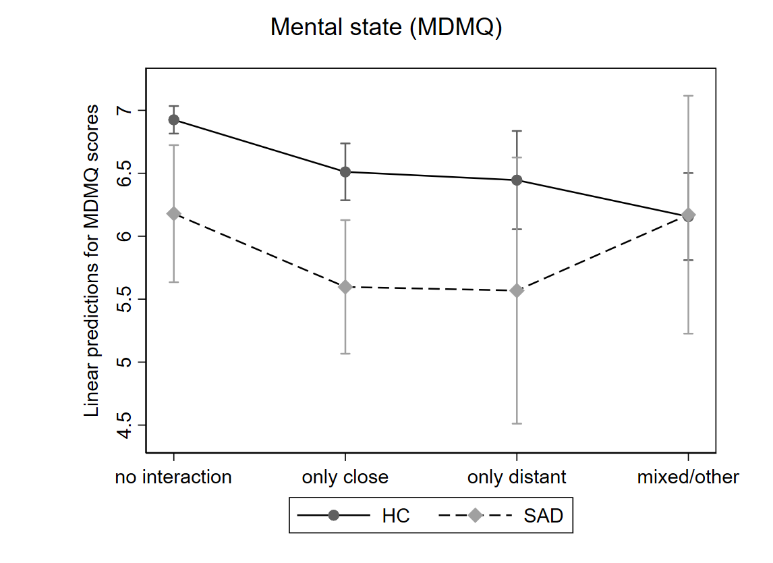


*Online Resource 4.* Predictive margins based on multilevel regression models analyzing the effects of negative social interactions on depression, anxiety, and mental state. Parts 1a-1c show predicted depression (1a) anxiety (1b) and mental state (1c) scores related to interaction behaviors for healthy controls (HC) and participants meeting the criteria for 12-months social anxiety disorder (SAD). Parts 2a-2c show predicted depression (2a) anxiety (2b) and mental state (2c) scores for the HC and SAD groups considering the interaction partner. Depression and anxiety scores have been logarithmized. Error bars indicate 95% confidence interval.
